# Plasma Circulating Mirnas Profiling for Identification of Potential Breast Cancer Early Detection Biomarkers

**DOI:** 10.31557/APJCP.2021.22.5.1375

**Published:** 2021-05

**Authors:** A. Rashid Jusoh, Sivanesan Vijaya Mohan, Tan Lu Ping, Tengku Ahmad Damitri Al Astani Bin Tengku Din, Juhara Haron, Roslini Che Romli, Hasnan Jaafar, Siti Norasikin Nafi, Tuan Ismail Tuan Salwani, Maya Mazuwin Yahya

**Affiliations:** 1 *Department of Biomedicine, School of Health Sciences, Universiti Sains Malaysia, Kelantan, Malaysia. *; 2 *Molecular Pathology Unit, Cancer Research Centre, Institute for Medical Research, Ministry of Health, Kuala Lumpur, Malaysia. *; 3 *Department of Chemical Pathology, School of Medical Sciences, Universiti Sains Malaysia, Kelantan, Malaysia.*; 4 *Department of Pathology, School of Medical Sciences, Universiti Sains Malaysia, Kelantan, Malaysia. *; 5 *Breast Cancer Awareness and Research Unit, Hospital Universiti Sains Malaysia, Kelantan, Malaysia. *; 6 *Department of Surgery, School of Medical Sciences, Universiti Sains Malaysia, Kelantan, Malaysia. *

**Keywords:** Breast cancer, miRNAs, biomarkers, PCR array, Hospital USM

## Abstract

**Objective:**

This study aimed to characterize the miRNA expression profiles from plasma samples of our local breast cancer patients in comparison to healthy control by using miRNA PCR Array.

**Methods:**

In this study, plasma miRNA profiles from eight early-stage breast cancer patients and nine age-matched (± 2 years) healthy controls were characterized by miRNA array-based approach, followed by differential gene expression analysis, Independent T-test and construction of Receiver Operating Characteristic (ROC) curve to determine the capability of the assays to discriminate between breast cancer and the healthy control.

**Results:**

Based on the 372-miRNAs microarray profiling, a set of 40 differential miRNAs was extracted regarding to the fold change value at 2 and above. We further sub grouped 40 miRNAs of breast cancer patients that were significantly expressed at 2-fold change and higher. In this set, we discovered that 24 miRNAs were significantly upregulated and 16 miRNAs were significantly downregulated in breast cancer patients, as compared to the miRNA expression of healthy subjects. ROC curve analysis revealed that seven miRNAs (miR-125b-5p, miR-142-3p, miR-145-5p, miR-193a-5p, miR-27b-3p, miR-22-5p and miR-423-5p) had area under curve (AUC) value > 0.7 (AUC p-value < 0.05). Overlapping findings from differential gene expression analysis, ROC analysis, and Independent T-Test resulted in three miRNAs (miR-27b-3p, miR-22-5p, miR-145-5p). Cohen’s effect size for these three miRNAs was large with d value are more than 0.95.

**Conclusion:**

miR-27b-3p, miR-22-5p, miR-145-5p could be potential biomarkers to distinguish breast cancer patients from healthy controls. A validation study for these three miRNAs in an external set of samples is ongoing.

## Introduction

International Agency for Research on Cancer declared that 18.1 million new cancer cases and 9.6 million cancer deaths in 2018. Out of that, lung cancer is the most diagnosed and the main cause of death. It is followed closely by breast cancer in the second place. Breast cancer also declared as the most commonly diagnosed and leading cause of cancer death among females worldwide (Bray et al., 2018). Thus, urgent action from the public, clinicians, researchers and policymakers throughout the globe to curb this disease from keep spreading. 

In Malaysia, a total of 115,238 new cancer cases were diagnosed for the period of 2012-2016. The ten most common cancers among Malaysian were breast, colorectal, lung, lymphoma, nasopharynx, leukaemia, prostate, liver, cervix uteri and ovary (MNCR, 2019). Breast cancer and colorectal cancer are the most common cancer cases among female and male respectively. In female, 21,925 new breast cancer cases were recorded that contributed to the 19% of total cancer cases among females. When we compare to the report from Malaysian National Cancer Registry from period of 2007- 2011, breast cancer cases are still the highest cases among the female and shown two percent increment from that period. With these 10 years trend we can conclude that the incidence of breast cancer is also alarming among Malaysian females. Thus, more effective measures needs to be done to diagnose the condition early and commence treatment early.

Delay in seeking diagnosis among Malaysian women with breast cancer has been considered the major factor in poor survival of patients. Many of them had advanced breast cancer by the time of diagnosis (Norsa’adah et al., 2011). It was further supported with current data from Malaysian Study on Cancer Survival (MySCan) reported 5-year relative survival of female with breast cancers stages III and IV are 59.7 % and 23.3 % respectively as compare to stages I and II with 87.5 % and 80.7 % respectively (NCR, 2018). 

In current practice, triple assessments are the standard approach for the diagnosis of breast cancer. However, their use has limitation especially for early detection of cancer. Mammography is less sensitive in younger women due to increased breast density (Checka et al., 2012). Next is the biopsy, it is reliable to detect breast cancer but invasive and painful. Meanwhile, Magnetic Resonance Imaging (MRI) is sensitive for the detection of breast cancer but is expensive (Rankin, 2018). Thus, there is a need for a novel minimally invasive diagnostic approach to supplement those methods mentioned previously and improve the detection rates at a reasonable cost. 

In terms of using serum tumor markers in breast cancer, the carcinoembryonic antigen (CEA) and carbohydrate antigen 15-3 which belong to the MUC1 family are the most useful serum tumor markers in the patient with breast cancer (Kabel, 2017). They are beneficial in monitoring the response to therapy and for early detection of recurrence or metastasis. However, they lack sensitivity for low volume disease and lack of specificity. Therefore, we need to find another novel biomarker, with high sensitivity and specificity for the detection of breast cancer as early as stage one.

Based on the literature we found a short, single-strand RNA with 19-23 nucleotides called microRNA or miRNA as potential biomarkers for early detection of cancer. The function of these miRNAs is to control the posttranscriptional regulation of gene expression in a broad range of the biological system (Lim et al., 2005; Baek et al., 2008). Their expression is often dysregulated in cancer (Hamam et al., 2017). miRNAs signature can be used for the following purposes, determine prognosis (Yanaihara et al., 2006), response to drug therapy (Meng et al., 2006) and patient susceptibility to cancer and metastasis (Ma et al., 2007). As of the role of miRNA in breast cancer, Cuk et al.,(2013) independently enforcing the utility of miRNAs as minimal invasive and early detection markers for breast cancer (Cuk et al., 2013). M’hamed et al., (2015) suggested that the miR-10b, miR-26a and miR-153 has potential to be the biomarkers for triple negative breast cancer (TNBC) (M’hamed et al., 2015). In other studies, Serpico et al., (2014) and Van Schooneveld et al., (2012) found that various breast cancer subtypes exhibits different molecular miRNA signatures (Van Schooneveld et al., 2012; Serpico et al., 2014). Meanwhile, Van Schooneveld et al., 2015 found, Let-7c, miR-10a and Let-7f associated with luminal type A, miR- 18a, miR-135b, miR-93 and MiR-155 associated with basal type breast cancer. Even though many research groups focusing on application of microRNA in breast cancer especially for diagnostic purposes but little consistency with respect to circulating microRNA panels among research group (Haman et al., 2017). Thus, more research should be done in this area to gather more consistent results in development of new biomarker for early detection of breast cancer.

Up to date, miRNA profiling of Malaysian population is still limited especially Kelantanese. Therefore, this study helps to provide new information or establish miRNA profiles of Malaysian breast cancer patients. At the same time, it will help in searching for new method that can complement the currently available standard diagnosis as well as guide a more tailored treatment. 

## Materials and Methods

*Sample collection*

This study was approved by the Ethical Committee of the Health Campus Universiti Sains Malaysia in Kelantan (ethical no: USM/JEPeM/16050172). Eight blood samples from patients with pathologically confirmed breast cancer were collected at the Breast Cancer Awareness and Research Unit (BESTARI) Hospital Universiti Sains Malaysia (Hospital USM), Kubang Kerian, Kelantan, Malaysia. At the same center, nine blood samples as healthy control were collected from healthy female volunteers. These volunteers are free from history of cancer, lumps and not receiving blood transfusion for the past three months. All participants were Malaysian-born women who had given their written, informed consent to participate in this study. The calculation of sample size was done by using PS software based on the 2-mean formula. Eight samples size are needed from each group. The eight samples from each group it already enough to provided significant power of 0.8. Clinical information on breast cancer and healthy control detail were mentioned in Table 1.

*RNA extraction*

Blood samples were centrifuged at 2,500 rpm for 10 minutes at room temperature. Plasma was transferred to a new tube, centrifuged at 3,000 rpm for 10 minutes at room temperature, and then aliquoted into microcentrifuge tubes and kept at -80°C before RNA extraction. We isolated total RNA containing small RNA from 400 μl of plasma previously thawed on ice using a miRNAeasy kit (Qiagen, Valencia, CA, USA) according to the manufacturer’s protocol. The RNA was eluted with 50 ml of RNAse-free water and converting RNA to cDNA. The concentration of all RNA samples was quantified using NanoDrop 1000 (Thermo Scientific, Wilmington, DE, USA). 

*Reverse transcriptase*

cDNA was synthesized in 20 µl reaction by using the miScript II RT kit (Qiagen, Valencia,

CA, USA) according to the manufacturer’s protocol.

*miRNA microarray profiling*

miRNA profiling of eight breast cancers and nine healthy controls plasma samples were performed with miScript™ miRNA PCR Array (Qiagen, USA) as mention in the manufactural protocol. This array contains 372 miRNAs related to breast cancer. Expression data were normalized using cel-miR-39 as the internal control. Gene expression level was quantified using the Quantostudio 12K Flex Real-Time PCR system (Applied Biosystem, USA). The reaction was amplified for 15 min at 95°C followed by 40 cycles of 95°C for 15 s, 55°C for 30 s and 70°C for 30 s. All reaction was run in triplicate. Threshold cycle (Ct) values for each miRNA were recorded, miRNA with Ct value < 35 at least in one group either in breast cancer or healthy control sample was selected. The fold change of miRNA expression in each breast cancer sample relative to the average expression in normal control was calculated based on the threshold cycle (Ct) value using the 2^-ΔΔCT^ method. if the fold change was equal or more than two-fold miRNA was considered significantly express (Cuk et al., 2013). 

*Diagnostic potential of targeted miRNAs*

Receiver operator characteristics (ROC) curves analysis were performed to evaluate the diagnostic potential of miRNAs extracted from plasma of breast cancer patients and healthy controls. The discriminative power between tumor and non-tumor sample were depicted by area under the curve (AUC). The optimal sensitivity and specificity from ROC curves also were determined. miRNAs with area under the curves (AUC) ≥ 0.7 was considered the miRNA with good diagnostic value that manage to discriminate between tumor and non-tumor samples. ROC curves analysis was done using Statistical Package for Social Science (SPSS) latest version.

*Statistical analysis*

Statistical analysis was performed using Statistical Package for the Science Social (SPSS) version 24 (IBM^®^ SPSS Software, Armonk, New York, USA). The independent T-Test was performed to assess the significant difference between cancer and healthy control groups of normally distributed data. A p-value of less than 0.05 was deemed to be significant. 

## Results

*miRNA profiling plasma of eight breast cancer patient*

We analyzed plasma miRNA profiles of eight early stages of breast cancer patients as well as nine healthy controls. Based on molecular characterization, all patients were luminal A molecular subtypes. Patients was age-matched to healthy control (± 2 years). The median and range of ages of the patients were 50.5 and 37-58 years respectively, while they were 52.0 and 37-68 years for healthy controls (Table 1).

*40 miRNAs shown ≥ 2.0 fold change of expression in plasma of breast cancer patient as compared to healthy control*

After normalization of raw array data and filtering, a total of 40 variants of miRNAs remain for statistical analysis. That 40 miRNAs shown higher expression with a fold change of ≥ 2.0 (Table 2). From that 40 miRNAs, 24 are upregulated and 16 downregulated. The upregulated and downregulated miRNAs were further analyzed by using Independent T-Test to find the significant difference between cancer and healthy control groups. We found that only three miRNAs shown significantly different with the following p-value, miR-27b-3p (p= 0.01), miR-22-5p (p= 0.02) and miR-145-5p (p= 0.04) (Table 2). The Cohen’s effect size of these three miRNAs also large with 1.13, 1.14 and 1.55 respectively.

*Diagnostic potential of circulating miRNAs using Receiver Operating Characteristic (ROC) analysis*

**Figure 1:**
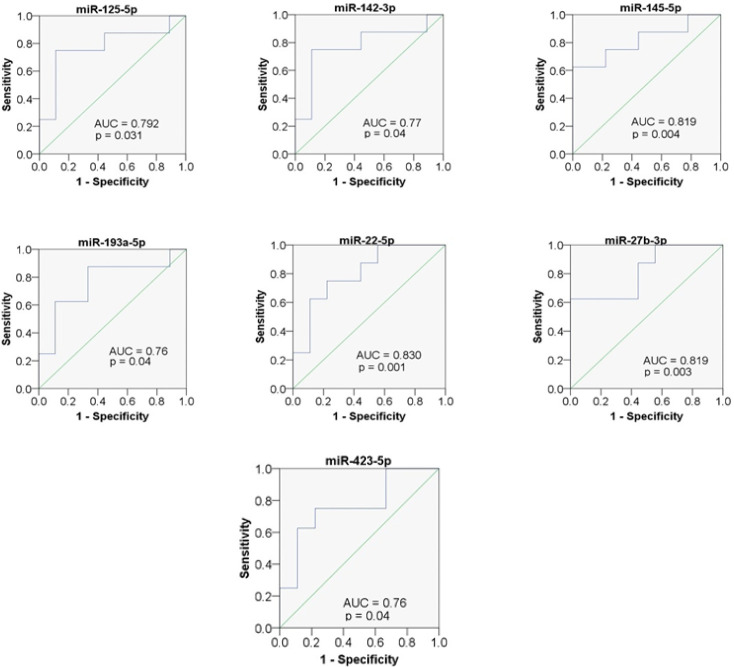
Area Under Curve of Receiver Operating Characteristic (ROC) for miR-125-5p, miR-142-3p, miR-145-5p, miR-193a-5p, miR-22-5p, miR-27b-3p and miR423-5p

This analysis was done for the 40 miRNAs with fold change ≥ 2.0 to find the most sensitive biomarkers or miRNAs that can discriminate between breast cancer and healthy control. AUC was measured, those miRNAs with AUC value > 0.7 was considered as good potential biomarkers for early detection of breast cancer. From this analysis we found that seven miRNAs with AUC > 0.7 (AUC p-value < 0.05) (Table 2 and Figure 1). They are miR-125b-5p, miR-142-3p, miR-145-5p, miR-193a-5p, miR-27b-3p, miR-22-5p and miR-423-5p. At the optimal cutoff value 7.14 for miR-125b-5p, the sensitivity and specificity were 75% and 88.90%. At the cutoff 4.50 for miR- 142-3p, the sensitivity and specificity were 75% and 88.90. At the cutoff 6.10 for miR-145-5p, the sensitivity and specificity were 62.5% and 100%. At the cutoff 7.72 for miR-193a-5p, the sensitivity and specificity were 87.5% and 66.7%. At the cutoff 6.36 for miR-27b-3p, the sensitivity and specificity were 62.5% and 100%. At the cutoff 8.09 for miR-22-5p, the sensitivity and specificity were 75% and 88.9%. At the cutoff 5.09 for miR-423-5p, the sensitivity and specificity were 75% and 77.8%.

*3 miRNAs showed significant different in expression in plasma of breast cancer patients as compared to the healthy control sample.*

Out of 40 miRNAs that shown either upregulated or downregulated with fold change ≥ 2.0 and further analyzed with ROC analysis. We found that only three miRNAs that shown AUC value with > 0.7 (p < 0.05). These miRNAs were miR-27b-3p, miR-22-5p and miR-145-5p. Thus, these three miRNAs can be considered as the most suitable biomarkers to undergo a validation process.

**Table 1:** Clinical Information of Patient, Their Tumor Characteristic and Information of Healthy Control

Variables	Breast cancer	Healthy Control
Age, y		
Median age	50.5	52
Range	37-58	37-60
Gender, n (%)		
Female	8 (100%)	9 (100%)
Tumor Size (pT) ,n(%)		
pT1	3 (37.5%)	
pT2	5 (62.5%)	
Molecular subtypes, n(%)		
Luminal A	8 (100%)	

**Table 2:** Minus Delta delta Ct, Fold Change, Gene Regulation, Cohen’s Effect Size, p-value of Independent T-Test and ROC Analysis for 40 miRNAs

miRNA	Minus delta delta Ct (-ΔΔCt)	Fold change (2^-ΔΔCt^)	Regulation	Cohen's Effect size	p-value (-ΔCt BC vs -ΔCt HC)	AUC	95% CI of AUC	p-value (AUC)
miR-101-3p	1.547	2.92	up	0.92	0.24	0.61	0.34 - 0.82	0.45
**miR-125b-5p**	1.885	3.69	up	0.95	0.09	0.79	0.53 - 0.95	0.03
**miR-142-3p**	1.363	2.57	up	0.97	0.07	0.78	0.51 - 0.94	0.04
miR-142-5p	1.093	2.13	up	0.37	0.24	0.58	0.33 - 0.81	0.58
miR-145-3p	1.260	2.39	up	0.20	0.60	0.53	0.28 - 0.77	0.86
***miR-145-5p***	1.570	2.97	up	1.13*	0.04	0.82	0.56 - 0.96	0
miR-146a-5p	1.305	2.47	up	0.55	0.32	0.61	0.35 - 0.83	0.45
miR-146b-5p	1.960	3.89	up	0.74	0.10	0.74	0.47 - 0.92	0.11
miR-148a-3p	1.305	2.47	up	0.77	0.19	0.69	0.43 - 0.89	0.17
miR-151a-3p	1.018	2.03	up	0.30	0.33	0.65	0.39 - 0.86	0.28
**miR-193a-5p**	2.129	4.37	up	0.81	0.31	0.76	0.50 - 0.93	0.04
***miR-22-5p***	2.179	4.53	up	1.14*	0.02	0.83	0.58 - 0.97	0.00
***miR-27b-3p***	2.894	7.43	up	1.55*	0.01	0.82	0.56 - 0.96	0.00
miR-30a-3p	1.881	3.68	up	0.41	0.42	0.63	0.36 - 0.84	0.41
miR-338-3p	1.242	2.37	up	0.44	0.38	0.6	0.34 - 0.82	0.51
miR-373-5p	1.044	2.06	up	0.43	0.39	0.6	0.34 - 0.82	0.51
miR-423-3p	1.041	2.06	up	0.51	0.32	0.64	0.38 - 0.85	0.34
**miR-423-5p**	1.203	2.30	up	0.86	0.10	0.76	0.50 - 0.93	0.04
miR-454-3p	1.119	2.17	up	0.32	0.52	0.58	0.33 - 0.81	0.58
miR-497-5p	1.237	2.36	up	0.66	0.19	0.68	0.42 - 0.88	0.20
miR-605-5p	2.044	4.12	up	0.55	0.28	0.64	0.38 - 0.85	0.35
miR-652-3p	1.240	2.36	up	0.46	0.36	0.65	0.39 - 0.86	0.30
miR-885-5p	2.224	4.67	up	0.73	0.17	0.74	0.47 - 0.92	0.07
miR-181c-5p	1.503	2.83	up	0.77	0.14	0.72	0.46 - 0.91	0.10
miR-1193	-1.668	3.18	down	0.30	0.55	0.6	0.34 - 0.82	0.52
miR-1231	-1.003	2.00	down	0.16	0.75	0.58	0.33 - 0.81	0.58
miR-1260b	-1.265	2.40	down	0.51	0.35	0.61	0.35 - 0.83	0.48
miR-139-5p	-1.590	3.01	down	0.51	0.31	0.61	0.35 - 0.83	0.48
miR-144-5p	-1.009	2.01	down	0.48	0.35	0.64	0.38 - 0.85	0.34
miR-1539	-1.184	2.27	down	0.30	0.54	0.57	0.31 - 0.80	0.65
miR-28-3p	-1.392	2.62	down	0.41	0.41	0.58	0.33 - 0.81	0.58
miR-28-5p	-1.384	2.61	down	0.64	0.22	0.69	0.43 - 0.89	0.2
miR-30e-3p	-3.550	11.71	down	0.65	0.2	0.67	0.39 - 0.44	0.24
miR-31-5p	-1.603	3.04	down	0.44	0.38	0.63	0.36 - 0.84	0.41
miR-421	-1.526	2.88	down	0.53	0.31	0.63	0.36 - 0.84	0.4
miR-4274	-1.293	2.45	down	0.77	0.13	0.71	0.44 - 0.90	0.12
miRNA	Minus delta delta Ct (-ΔΔCt)	Fold change (2-ΔΔCt)	Regulation	Cohen's Effect size	p-value (-ΔCt BC vs -ΔCt HC)	AUC	95% CI of AUC	p-value (AUC)
miR-4689	-1.202	2.3	down	0.37	0.45	0.65	0.34 - 0.82	0.3
miR-93-3p	-2.196	4.58	down	0.5	0.32	0.68	0.53 - 0.95	0.2
miR-205-5p	-1.989	3.97	down	0.6	0.24	0.69	0.51 - 0.94	0.16
miR-219a-1-3p	-1.004	2.01	down	0.26	0.6	0.63	0.33 - 0.81	0.43

*, Cohen’s effect size > 1.13; CI, confident interval, miRNAs with bolded representing the miRNAs with AUC value more than 0.7( p-value < 0.05), miRNAs with italic representing the miRNAs with AUC value more than 0.7 and p-value of independent T-Test with p < 0.05

## Discussion

Early detection of breast cancer is a major concern among the clinicians. Most of the patients came with late stages of cancer that delay the treatment program (Norsa’adah et al., 2011). In this study, we want to identify the potential biomarkers called miRNA that can detect breast cancer at an earlier stage with the minimally invasive technique. miRNA could be detected in the circulation of the cancer patient and has been suggested in several studies to reflect breast cancer progression (Cuk et al., 2013; Mangolini et al., 2015; Hamam et al., 2016). We had investigated the expression of miRNA extracted from blood plasma of the early stages of the breast cancer patients and healthy controls using the miScript PCR System. The system is one of the quantitative RT-PCR platforms, a platform reported to have sensitivity and robustness superior to other widely available platforms especially in the lower concentration range of RNA which is critical for the study of cell-free microRNA (Mangolini et al., 2015). Through the miScript PCR System, we studied the expression of 372 variants of miRNA that can potentially serve as a useful molecular marker for heart and liver injury or disease like atherosclerosis, diabetes and organ-specific cancers. We found that only 40 miRNAs showed higher expression with a fold change of ≥ 2.0 (Table 2), from that numbers 24 are upregulated and 16 downregulated. This result is similar to the study done by Zhao et al., (2012), where they found 31 miRNAs were differentially expressed in Caucasian American and 18 miRNAs differentially expressed in African Americans. However, only two miRNAs overlapped between that two races. When we compared with our results, we found that only miR-27b overlapped with Caucasian American breast cancer patients. 

That 24 upregulated and 16 downregulated miRNAs were further analyzed by using ROC analysis to determine the most sensitive miRNAs that can discriminate between breast cancer patients and healthy controls. We found seven miRNAs with AUC > 0.7 (p- value < 0.05) as stated in Table 2. They were miR-125b-5p, miR-142-3p, miR-145-5p, miR-193a-5p, miR-27b-3p, miR-22-5p, miR-423-5p. miR-125b-5p and miR-145-5p were upregulated or showed higher expression in breast cancer patients as compared to healthy controls. These findings were similar to the study done by Mar-Aguilar et al., (2013) and Wang et al., (2012). They found significant expression of miR-125b in the serum of breast cancer patients as compared to healthy controls. In our study, miR-142-3p of breast cancer patients were significantly higher as compared to the healthy subjects. Our finding is contradicted with the trend observed in a previous study (Mansoori et al., 2019). Their study has found that miR-142-3p was downregulated in human breast cancer cell lines. The above trends describe that the different population may have different pattern of miRNA profiles. They also concluded that, the induction of miR-142-3p could regulate important tumor suppressor miRNAs in breast cancer cells. It has been reported that the downregulation of miR-142-3p attenuates breast cancer stem cell characteristic and reduce radiotherapy resistance (Troschel et al., 2018). miR-193a-5p has shown higher expression in breast cancers as compared to healthy controls. These findings contradicted the study done by Xie et al., (2017), where they found decreasing expression of miR-193a-5p in breast cancer cell lines and breast cancer tissues. They also found that miR-193a-5p inhibits breast cancer proliferation and metastasis by downregulation *WT1* gene. The differences might be approved by the different type of sample and patient origins used in both studies. However, Pigati et al., (2010), stated that releases of miRNAs do not necessarily reflect the abundance of miRNA in the cell of origin. It has higher expression in tissue and lower expression in blood circulation or vice versa. miR-27b-3p has shown higher expression in breast cancer patients plasma as compared to healthy controls. This finding is supported by a study done by Hannafon et al., (2019), where dysregulation of miR-27b is a significant driving force behind the breast cancer progression. Lastly, miR-22-5p and miR-423-5p also had shown higher expression in breast cancer plasma as compared to healthy controls. To our knowledge, none of the studies reported these two miRNAs associates with breast cancer. However, one study reported this miRNA is associated with expression of the long non-coding RNA MEG3 in acute myeloid leukemia (Yao et al., 2017). Meanwhile, other studies reported that miR-423-5p is associated with gastric cancer and ovarian cancer (Tang et al., 2018; Yang et al., 2018). 

Further analysis against these 40 miRNAs using independent T-Test, we identified three miRNAs (miR-27b-3p, miR-22-5p and miR-145-5p) that were significantly expressed in breast cancers as compared to healthy controls. That result is overlapping with ROC analysis where these three miRNAs had shown the highest AUC value and good sensitivity and specificity value as compared to others. 

In conclusion, we identified three miRNAs (miR-27b-3p, miR-22-5p and miR-145-5p) that can be considered as the most suitable biomarkers to undergo a validation process. However, the potential of other four miRNAs (miR-125b-5p, miR-142-3p, miR-193a-5p and miR-423-5p) that having AUC value > 0.7 should not be ignored. Currently our team is in the process of validating the above miRNAs via qRT-PCR method to confirm the expression of that miRNAs in larger sample size. We also plan to involve the sample with tumor size as early as T1 until T4. Furthermore, we plan to compare the expression of miRNAs in blood and tissue of breast cancer patients.

## Author Contribution Statement

The authors confirm contribution to the paper as follows: study conception and design: TAD, MMY, HJ, TSTI and JH; data collection: TAD, MMY, RR, SVM and TLP; analysis and interpretation of results: TLP, ARJ and SNN; draft manuscript preparation: ARJ and TAD. All authors reviewed the results and approved the final version of the manuscript.

## References

[B1] Baek D, Villén J, Shin C (2008). The impact of microRNAs on protein output. Nature.

[B2] Bray F, Ferlay J, Soerjomataram I (2018). Global cancer statistics 2018: GLOBOCAN estimates of incidence and mortality worldwide for 36 cancers in 185 countries. CA Cancer J Clin.

[B3] Checka CM, Chun JE, Schnabel FR (2012). The relationship of mammographic density and age: implications for breast cancer screening. Am J of Roenigenol.

[B4] Cuk K, Zucknick M, Heil J (2013). Circulating microRNAs in plasma as early detection markers for breast cancer. Int J Cancer.

[B5] Hamam R, Ali AM, Alsaleh KA (2016). microRNA expression profiling on individual breast cancer patients identifies novel panel of circulating microRNA for early detection. Sci Rep.

[B6] Hamam R, Hamam D, Alsaleh KA (2017). Circulating microRNAs in breast cancer: novel diagnostic and prognostic biomarkers. Cell Death Dis.

[B7] Hannafon BN, Cai A, Calloway CL (2019). miR-23b and miR-27b are oncogenic microRNAs in breast cancer: evidence from a CRISPR/Cas9 deletion study. BMC Cancer.

[B8] Kabel AM (2017). Tumor markers of breast cancer: New prospectives. J Oncol Sci.

[B9] Lim LP, Lau NC, Garrett-Engele P (2005). Microarray analysis shows that some microRNAs downregulate large numbers of target mRNAs. Nature.

[B10] M’hamed IF, Privat M, Ponelle F (2015). Identification of miR-10b, miR-26a, miR-146a and miR-153 as potential triple-negative breast cancer biomarkers. Cell Oncol.

[B11] Ma L, Teruya-Feldstein J, Weinberg RA (2007). Tumour invasion and metastasis initiated by microRNA-10b in breast cancer. Nature.

[B12] Mangolini A, Ferracin M, Zanzi MV (2015). Diagnostic and prognostic microRNAs in the serum of breast cancer patients measured by droplet digital PCR. Biomark Res.

[B13] Mar-Aguilar F, Mendoza-Ramírez JA, Malagón-Santiago I (2013). Serum circulating microRNA profiling for identification of potential breast cancer biomarkers. Dis Markers.

[B14] Meng F, Henson R, Lang M (2006). Involvement of human micro-RNA in growth and response to chemotherapy in human cholangiocarcinoma cell lines. Gastroenterology.

[B17] Norsa’adah B, Rampal KG, Rahmah MA (2011). Diagnosis delay of breast cancer and its associated factors in Malaysian women. BMC Cancer.

[B18] Pigati L, Yaddanapudi SC, Iyengar R (2010). Selective release of microRNA species from normal and malignant mammary epithelial cells. PLoS One.

[B19] Rankin SC (2018). MRI of the breast. Br J Radiol.

[B20] Serpico D, Molino L, Di Cosimo S (2014). microRNAs in breast cancer development and treatment. Cancer Treat Rev.

[B21] Tang X, Zeng X, Huang Y (2018). miR4235p serves as a diagnostic indicator and inhibits the proliferation and invasion of ovarian cancer. Exp Therap Med.

[B22] Troschel FM, Böhly N, Borrmann K (2018). miR-142-3p attenuates breast cancer stem cell characteristics and decreases radioresistance in vitro. Tumor Biol.

[B23] Van Schooneveld E, Wouters MC, Van der Auwera I (2012). Expression profiling of cancerous and normal breast tissues identifies microRNAs that are differentially expressed in serum from patients with (metastatic) breast cancer and healthy volunteers. Breast Cancer Res.

[B24] Wang H, Tan G, Dong L (2012). Circulating MiR-125b as a marker predicting chemoresistance in breast cancer. PLoS One.

[B25] Xie FY, Hosany S, Zhong S (2017). MicroRNA-193a inhibits breast cancer proliferation and metastasis by downregulating WT1. PLoS One.

[B26] Yanaihara N, Caplen N, Bowman E (2006). Unique microRNA molecular profiles in lung cancer diagnosis and prognosis. Cancer Cell.

[B27] Yang H, Fu H, Wang B (2018). Exosomal miR-423-5p targets SUFU to promote cancer growth and metastasis and serves as a novel marker for gastric cancer. Mol Carcinogenesis.

[B28] Yao H, Sun P, Duan M (2017). microRNA-22 can regulate expression of the long non-coding RNA MEG3 in acute myeloid leukemia. Oncotarget.

[B29] Zhao H, Shen J, Medico L (2010). A pilot study of circulating miRNAs as potential biomarkers of early stage breast cancer. PLoS One.

